# Development of automated brightfield double *In Situ *hybridization (BDISH) application for *HER2 g*ene and chromosome 17 centromere (CEN 17) for breast carcinomas and an assay performance comparison to manual dual color *HER2 *fluorescence *In Situ *hybridization (FISH)

**DOI:** 10.1186/1746-1596-3-41

**Published:** 2008-10-22

**Authors:** Hiroaki Nitta, Beatrice Hauss-Wegrzyniak, Megan Lehrkamp, Adrian E Murillo, Fabien Gaire, Michael Farrell, Eric Walk, Frederique Penault-Llorca, Masafumi Kurosumi, Manfred Dietel, Lin Wang, Margaret Loftus, James Pettay, Raymond R Tubbs, Thomas M Grogan

**Affiliations:** 1 Office of Medical Affairs, Ventana Medical Systems, Inc, Tucson, AZ, USA; 2 Advanced Staining, Ventana Medical Systems, Inc, Tucson, AZ, USA; 3 Discovery, Ventana Medical Systems, Inc, Tucson, AZ, USA; 4 Département de Pathologie, Centre Jean Perrin, Clermont-Ferrand Cédex, France; 5 Pathology and Laboratory Medicine Institute, Saitama Cancer Center, Saitama, Japan; 6 Institute of Pathology, Charité-University Medicine Berlin, Berlin, Germany; 7 Pathology and Laboratory Medicine Institute, Cleveland Clinic Foundation, Cleveland, OH, USA; 8 The Cleveland Clinic Lerner College of Medicine, Cleveland, OH, USA; 9 Department of Pathology, College of Medicine, the University of Arizona, Tucson, AZ, USA

## Abstract

**Background:**

Human epidermal growth factor receptor 2 (*HER2*) fluorescence *in situ *hybridization (FISH) is a quantitative assay for selecting breast cancer patients for trastuzumab therapy. However, current *HER2 *FISH procedures are labor intensive, manual methods that require skilled technologists and specialized fluorescence microscopy. Furthermore, FISH slides cannot be archived for long term storage and review. Our objective was to develop an automated brightfield double *in situ *hybridization (BDISH) application for *HER2 *gene and chromosome 17 centromere (CEN 17) and test the assay performance with dual color *HER2 *FISH evaluated breast carcinomas.

**Methods:**

The BDISH assay was developed with the nick translated dinitrophenyl (DNP)-labeled *HER2 *DNA probe and DNP-labeled CEN 17 oligoprobe on the Ventana BenchMark^® ^XT slide processing system. Detection of *HER2 *and CEN 17 signals was accomplished with the silver acetate, hydroquinone, and H_2_O_2 _reaction with horseradish peroxidase (HRP) and the fast red and naphthol phosphate reaction with alkaline phosphatise (AP), respectively. The BDISH specificity was optimized with formalin-fixed, paraffin-embedded xenograft tumors, MCF7 (non-amplified *HER2 *gene) and BT-474 (amplified *HER2 *gene). Then, the BDISH performance was evaluated with 94 routinely processed breast cancer tissues. Interpretation of *HER2 *and CEN 17 BDISH slides was conducted by 4 observers using a conventional brightfield microscope without oil immersion objectives.

**Results:**

Sequential hybridization and signal detection for *HER2 *and CEN 17 ISH demonstrated both DNA targets in the same cells. *HER2 *signals were visualized as discrete black metallic silver dots while CEN 17 signals were detected as slightly larger red dots. Our study demonstrated a high consensus concordance between *HER2 *FISH and BDISH results of clinical breast carcinoma cases based on the historical scoring method (98.9%, Simple Kappa = 0.9736, 95% CI = 0.9222 – 1.0000) and the ASCO/CAP scoring method with the FISH equivocal cases (95.7%, Simple Kappa = 0.8993%, 95% CI = 0.8068 – 0.9919) and without the FISH equivocal cases (100%, Simple Kappa = 1.0000%, 95% CI = 1.0000 – 1.0000).

**Conclusion:**

Automated BDISH applications for *HER2 *and CEN 17 targets were successfully developed and it might be able to replace manual two-color *HER2 *FISH methods. The application also has the potential to be used for other gene targets. The use of BDISH technology allows the simultaneous analyses of two DNA targets within the context of tissue morphological observation.

## Background

The human epidermal growth factor receptor 2 (*HER2*) oncogene, located on the long arm of chromosome 17 (17q12-q21), is over-expressed or amplified in approximately 20% of breast carcinoma cases [1,2]. *HER2 *status in breast cancer is used as a prognostic factor, a predictive factor, and a therapy selection factor [3] for the humanized monoclonal antibody trastuzumab (Herceptin^®^; Genentech), which is an FDA approved drug for use as monotherapy or combined chemotherapy for treatment of breast cancer patients with amplified *HER2 *status. Trastuzumab adjuvant treatment for early *HER2 *positive breast cancer is effective for improving patient survival and cost-effectiveness analyses of such treatment have shown acceptable ratios [4-7]. However, there is a negative aspect to trastuzumab therapy, namely cardiac toxicity [3], which is possibly due to myocardial *HER2 *gene over-expression associated with anthracycline treatment [8] and substantial cost.

Quantitative *HER2 *fluorescence *in situ *hybridization (FISH) analyses for detecting *HER2 *gene amplification and semi-quantitative HER2 immunohistochemistry (IHC) analyses for detecting over-expressed HER2 protein are performed to determine the *HER2 *status of breast cancer patients. The optimal scoring method for determination of *HER2 *gene status is the use of chromosome 17 centromere (CEN 17) enumeration for calculating the *HER2*/CEN 17 ratio [9]. One study showed that chromosome 17 polysomy (13%) and chromosome 17 monosomy (2%) were confirmed among 147 breast cancer cases with 2+ and 3+ HER2 IHC scores [10]. Also, chromosome 17 polysomy is a key prognosis indicator for breast cancer patients. Patients with chromosome 17 polysomy and no *HER2 *gene amplification have better prognosis compared to patients with *HER2 *gene amplification [11]. Dual color FISH for *HER2 *and CEN 17 targets is recommended especially for borderline IHC cases [12]. However, there are additional drawbacks to conducting *HER2 *FISH assays beyond the requirement for a specialized fluorescence microscope and the difficulty of preserving FISH signal during a long term storage. For example, *HER2 *FISH testing has exhibited a higher assay failure rate in the hands of some investigators when compared to HER2 IHC testing (5% *vs*. 0.08%), the FISH assay procedure time is longer than the IHC assay (36 hours *vs*. 4 hours), and the FISH interpretation time is longer than IHC interpretation time (7 minutes *vs*. 45 seconds) [1]. Another disadvantage of the FISH assay is the difficulty of correlating cytomorphological aspects of the tissue sample with the gene status [13]. Furthermore, tissue slides for the dual color FISH test are still processed manually in most laboratories, which practice can introduce human errors during the lengthy assay. In fact, FISH assays may not always be performed accurately [14]. An international *HER2 *proficiency testing study showed that there was 20% (4 out of 20 samples) discordance with *HER2 *FISH testing among 5 experienced laboratories [15]. On the other hand, some reports using proficiency testing surveys conducted by the College of American Pathologists have demonstrated a much higher concordance for FISH [16].

There are alternatives to FISH for determining *HER2 *gene status. The chromogenic *in situ *hybridization (CISH) assay using a DAB chromogen and H_2_O_2 _substrate system for horseradish peroxidase (HRP)-based signal detection has been evaluated and the value of this assay for assessing *HER2 *status has been demonstrated [12,17-22]. CISH slides can be interpreted using an ordinary brightfield microscope without oil-immersion lenses and can provide visible tissue morphology for correlation with the *HER2 *gene signal. However, with the current CISH method, the assessment of the *HER2*/CEN 17 ratio is conducted by enumerating *HER2 *and CEN 17 separately using two different tissue sections.

Other brightfield microscopy *in situ *hybridization (ISH) methods use autometallography and enzyme metallography: 1) Nanogold^® ^with gold enhancement *in situ *hybridization (GOLDFISH) [23,24] and 2) enzyme metallography or silver *in situ *hybridization (SISH) [25-27]. The GOLDFISH procedure utilizes the tyramide signal amplification principle and produces large clusters for amplified *HER2 *gene signal. On the other hand, the SISH method produces discrete metallic silver black signals. Horseradish peroxidase (HRP) of the detection system reacts with silver acetate, hydroquinone, and H_2_O_2 _and deposits metallic silver particles at the reaction site. The reaction product can be seen as discrete black dots under a brightfield microscope. Advantages of SISH include the high sensitivity for detection of single gene copies, the high resolution for quantifying DNA targets, and the high contrast with tissue counterstaining for visual separation of the signal and tissue morphology [27].

Recently, an automated *HER2 *SISH assay was evaluated for assessing the inter-observer interpretative reproducibility of the *HER2 *gene status of 99 clinical cases when compared against the reference standard FISH results [28]. Overall concordance between dual color *HER2 *FISH and single color *HER2 *SISH was 96.0% (kappa = 0.754, 95% CI = 0.518–0.993) and the discrepancies were mainly observed among tumors with the heterogeneity of tumor cell populations [28]. Advantages of the SISH assay compared to the CISH assay include that the SISH assay produces signal clusters and separately visualized discrete black dots that are easier to count in the majority of clinical samples. With SISH, the endogenous gene copies present in non-neoplastic stromal cells are also routinely and reproducibly visualized. However, like the CISH assay, the detection of CEN 17 signal cannot be performed on the same tissue section. Thus, it would be ideal to visualize both *HER2 *and CEN 17 on the same tissue section like two-color FISH assays. Dual ISH staining for *HER2 *gene and CEN 17 would be beneficial for analyzing chromosome 17 aneusomy and for delineation of cases displaying genotypic intratumoral heterogeneity.

One prerequisite for testing *HER2 *status reproducibly is the use of automation for conducting the test in the same manner among different laboratories located in different parts of the world. Thus, as a step toward standardizing *HER2 *testing, our objective was to develop an automated brightfield double *in situ *hybridization (BDISH) assay for simultaneous detection of *HER2 *and CEN 17 DNA targets on formalin-fixed, paraffin-embedded breast cancer tissue samples. Using this method, *HER2 *status testing can be conducted in a simplified manner for more accurately identifying the patients who are eligible for trastuzumab therapy and potentially leading to the improvement of breast cancer patient care in the future.

## Methods

### Tissue samples

MCF7 and BT-474 xenograft tumors were utilized for optimizing the BDISH assay. MCF7 is a breast adenocarcinoma cell line with non-amplified *HER2 *status and BT-474 is a breast ductal carcinoma with amplified *HER2 *status (50–60 copies of *HER2*) and chromosome 17 polysomy [29]. Paraffin sections (4 μm) containing tissue cores of formalin-fixed, paraffin-embedded MCF7 and BT-474 xenograft tumors were placed onto Superfrost^® ^Plus glass slides (Erie Scientific Company, Portsmouth, New Hampshire).

Ninety-four (94) breast cancer cases were used from the Cleveland Clinic Foundation and the Cleveland Clinic Lerner College of Medicine, Cleveland, OH, USA under IRB approved protocol. Tissue samples were routinely processed for paraffin-embedding after fixing with an alcoholic formalin fixative. All breast cancer cases had been previously tested for *HER2 *status by FISH using the PathVysion^® ^HER-2 DNA Probe Kit (Abbott Molecular, Des Plaines, Illinois) at the Cleveland Clinic Foundation. However, it should be noted that non-consecutive tissue sections were used for FISH and BDISH analyses.

### Brightfield *in situ *hybridization

The BenchMark^® ^XT automated slide processing system (Ventana Medical Systems, Inc., Tucson, Arizona) was used for the optimization and performance evaluation of the BDISH assay for *HER2 *and CEN 17 DNA targets. A protocol was established so that the entire assay procedure consisting of baking, deparaffinization, pretreatment, hybridization, stringency wash, signal detection, and counterstaining was completed as a one-step fully automated assay. Paraffin tissue sections on glass slides were baked at 65°C for 20 minutes prior to the deparaffinization step with EZ Prep™ (Ventana) at 75°C for 16 minutes. Deparaffinized tissue sections were pretreated with a combination of heat treatment with Reaction Buffer (Tris-based pH 7.6 solution, Ventana) and ISH Protease 2 or ISH Protease 3 (Ventana) to unmask DNA targets. Pretreatment conditions were chosen for optimal signal to noise ratio and tissue morphology preservation for the xenograft control slides as well as clinical case tissue slides.

**Figure 1:**
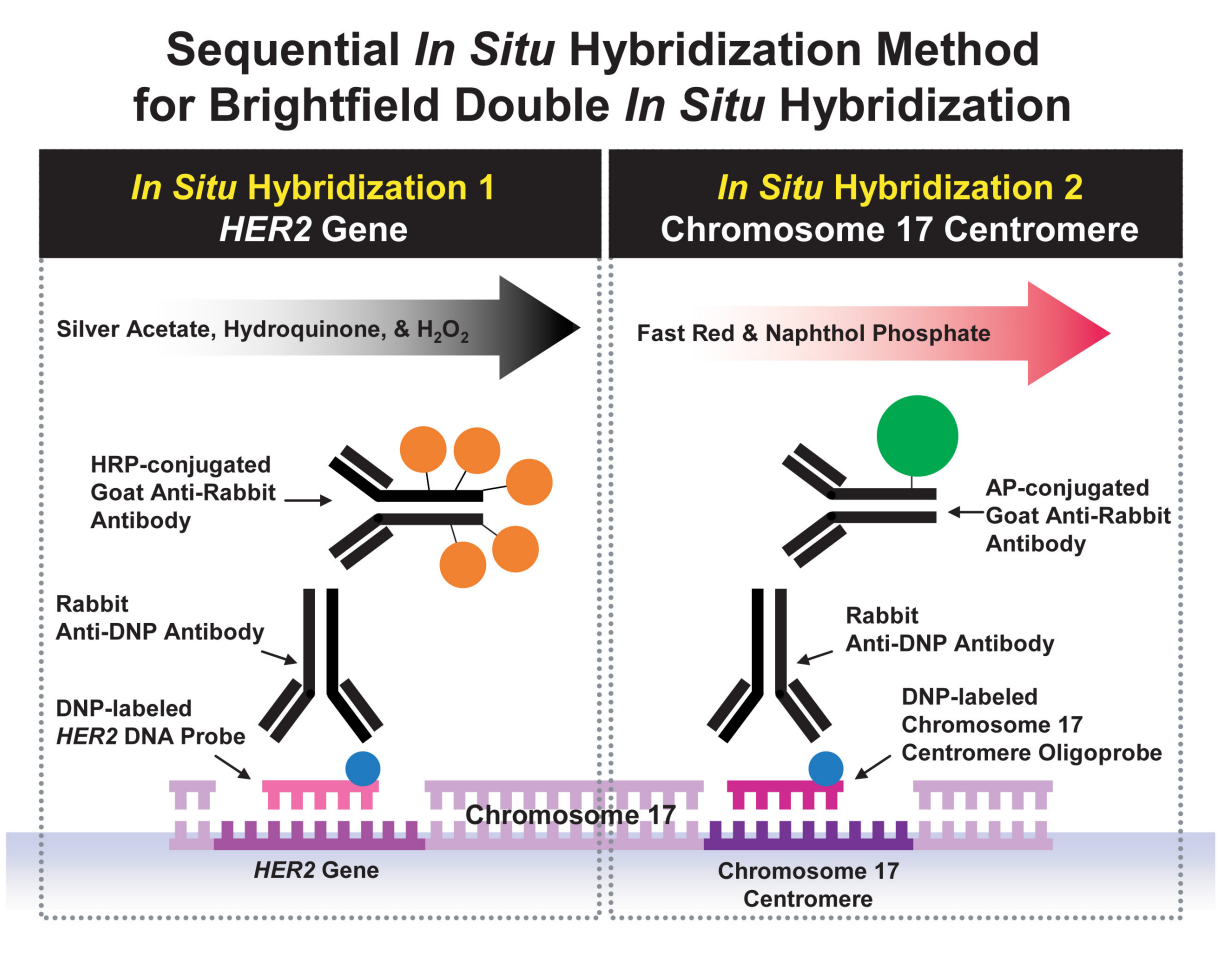
**Brightfield double *in situ *hybridization (BDISH) signal detection scheme with a sequential *in situ *hybridization method**. *HER2 *gene signal was detected with a DNP-labeled nick translated DNA probe hybridization followed by silver signal detection system (silver acetate, hydroquinone, and H_2_O_2 _reaction). Then, chromosome 17 centromere (CEN 17) signal was detected with a DNP-labeled CEN 17 oligoprobe hybridization followed by fast red and naphthol phosphate reaction signal detection system.

Sequential ISH procedures for *HER2 *and CEN 17 signal detection were conducted for a complete BDISH assay (Figure 1). Reaction Buffer was used for the washing steps during immunological detection. Liquid Coverslip™ (LCS, a hydrophobic reagent, Ventana) was used for controlling liquid evaporation throughout the assay. For *HER2 *gene detection, the INFORM^® ^*HER2 *DNA Probe (Ventana), a dinitrophenyl (DNP)-labeled, nick-translated repeat deleted DNA probe was applied to the glass slide for co-denaturing the probe and target at 95°C. Then, the hybridization step was conducted at 52°C for 2 hours. After 3 stringency wash steps were performed at 72°C with 2× SCC (Ventana), tissue sections were incubated with monoclonal rabbit anti-DNP antibody (Ventana) for 20 minutes and then with HRP-conjugated anti-rabbit antibody for 16 minutes at 37°C. The metallic silver deposit for *HER2 *ISH signal was developed using silver acetate, hydroquinone, and H_2_O_2 _reaction in the presence of HRP using the *ultra*View™ SISH Detection Kit (Ventana). For CEN 17 detection, the INFORM Chromosome 17 Probe (Ventana), a DNP-labeled oligoprobe, was applied to the tissue sections, denatured at 95°C and hybridized at 44°C for 2 hours. Then, after 3 stringency wash steps at 59°C with 2× SSC, tissues were incubated with rabbit monoclonal anti-DNP antibody for 20 minutes and then with an alkaline phosphatase (AP)-conjugated anti-rabbit antibody for 12 minutes at 37°C. Finally, the signal for CEN 17 was visualized with a fast red and naphthol phosphate reaction using *ultra*View Red ISH Detection Kit. Diaminobenzidine (DAB) chromogen and H_2_O_2 _substrate reagents from the *ultra*View Universal DAB Detection Kit (Ventana), 5-bromo-4 chloro-3-indolyl phosphate (BCIP) substrate and nitro blue tetrazolium (NBT) oxidant reagents from the ISH *i*VIEW™ Blue Detection Kit (Ventana), and a ready-to-use tetramethyl benzidine (TMB) solution (Fitzgerald Industries International, Concord, Massachusetts) were also evaluated for CEN 17 signal detection of the BDISH application. Because both *HER2 *and CEN 17 probes were labeled with the same DNP hapten, CEN 17 signal detection was completed without DNP-labeled CEN 17 probe after *HER2 *signal detection to ensure that the anti-DNP antibody of CEN 17 detection didn't recognize the DNP hapten of *HER2 *probe.

Single or double stained tissue sections for *HER2 *and/or CEN 17 targets were counterstained with Hematoxylin II (Ventana) for 4 or 8 minutes and Bluing Reagent (Ventana) for 4 minutes. Counterstained slides were first rinsed with distilled water containing DAWN^® ^(Proctor & Gamble Company, Cincinnati, Ohio) for removing LCS from slides and then rinsed with distilled water until soap was removed completely from the slide. Slides were blotted very gently with paper towels and completely dried at 45°C or 65°C in the oven for at least 15 minutes. One drop of Cytoseal™ 60 (Richard-Allen Scientific) was applied onto a dried slide and a glass coverslip was carefully placed onto the slide. Excess mounting media was removed from the slides by gently pressing the slides against paper towels. Different coverslipping methods were also evaluated for preserving the fast red staining during the assay development. BDISH results were observed with a Nikon ECLIPSE 90*i *microscope (Nikon Instruments Inc., Melville, New York) equipped with Nikon digital camera DXM1200F (Nikon) without oil immersion objective lenses, up to 60×. However, for presentation purposes, mainly comparing to FISH images taken with a 100× objective lens, brightfield photographs contained in this report were obtained using a 100× oil immersion objective lens.

### FISH

PathVysion HER-2 DNA Probe Kit was used for the FISH test for *HER2 *and CEN 17 targets of xenograft tumor controls as previously described [30]. Photographs of FISH images were taken with Zeiss Axioplan 2 microscope (Carl Zeiss MicroImaging, Inc., Thornwood, New York) with Metasystems JAIM4+ CCD1 Charge Coupling Imaging Camera (MetaSystems Group Inc., Watertown, Massachusetts) at 100× using an oil-immersion lens.

### BDISH performance test

Performance of the BDISH assay was compared to FISH results as the reference standard using the historical criteria for *HER2 *amplification with the PathVysion assay (Negative: *HER2*/CEN 17 < 2.0 and Positive: *HER2*/CEN 17 ≥ 2.0) and using the ASCO/CAP guideline criteria (Negative: *HER2*/CEN 17 > 1.8, Equivocal: 1.8 ≤ *HER2*/CEN 17 ≤ 2.2, and Positive: *HER2*/CEN 17 > 2.2) with or without the equivocal cases. Scoring BDISH slides was conducted by 4 observers (MK, MD, FPL, and RRT), who were experienced with scoring *HER2 *FISH slides. Scoring occurred at different sites and at different occasions using different microscopes. Each individual observer evaluated the set of slides at their own pace and judgement. No scores were provided by the observers when the staining quality was deemed not adequate. There was no communication among observers regarding their scoring experience of the BDISH slides. Concordance data of FISH scores *vs*. consensus BDISH scores among 4 observers and FISH scores *vs*. individual BDISH scores by 4 observers were determined using SAS 9.1 (SAS Institute Inc, Cary, North Carolina) in calculating frequency tables and Kappa statistics. The consensus among observers was defined as the agreement of three or more observers on a given observation. Scoring of BDISH assays was also analyzed for the sensitivity and specificity against FISH scores with the historical scoring method and the ASCO/CAP scoring method without the equivocal cases. Discordant cases were investigated by a non-observer (HN) for possible causes using BDISH slides.

## Results

### BDISH assay optimization

**Figure 2:**
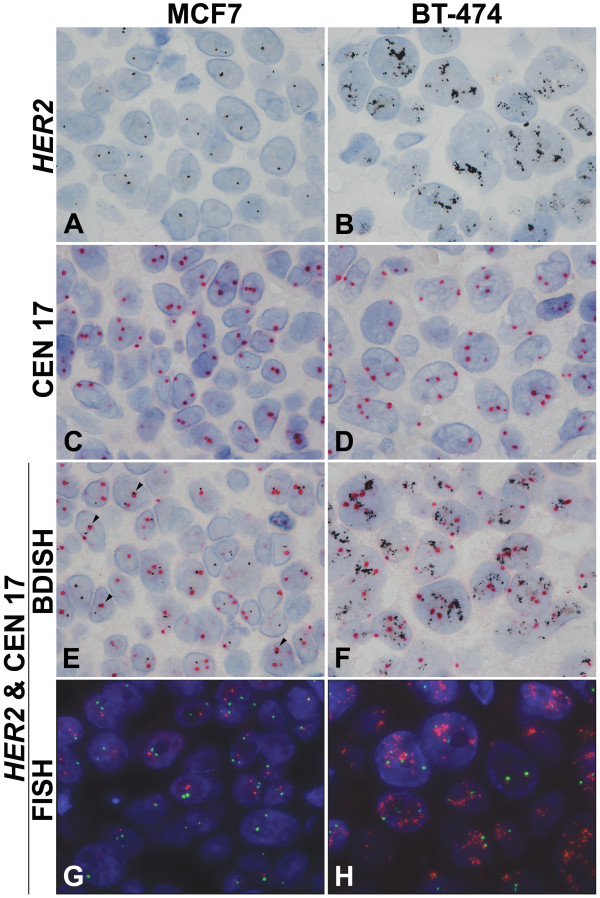
**Brightfield *in situ *hybridization and dual color fluorescence *in situ *hybridization (FISH) for *HER2 *and CEN 17**. *HER2 *and CEN 17 detection with formalin-fixed, paraffin-embedded xenograft tumors, MCF7 (non-amplified *HER2 *gene and chromosome 17 polysomy) (A, C, E, G) and BT-474 (amplified *HER2 *gene and chromosome 17 polysomy) (B, D, F, H). Normal *HER2 *gene signal is seen as black dots in the nuclei of MCF7 xenograft tumor (A) while amplified *HER2 *gene signal is seen as clusters of black dots in the nuclei of BT-474 tumor (B). CEN 17 signal is detected as red dots that are slightly larger than silver black dots (C, D). Double staining of *HER2 *gene and CEN 17 is obtained with silver grains and red dots (E, F). Individual *HER2 *gene and CEN 17 signals can be still recognized when both targets are co-localized (arrow heads, E). *HER2 *FISH signal is red-orange and CEN 17 FISH signal is green in the blue nuclei counterstained with DAPI (G, H). 100×.

Images of *HER2 *single ISH, CEN 17 single ISH, and *HER2 *and CEN 17 BDISH results with formalin-fixed, paraffin-embedded xenograft tumor sections are presented in Figure 2. Single copies of *HER2 *signal were recognized as black discrete dots in the nuclei with MCF7 xenograft tumor (Figure 2A) while amplified *HER2 *gene signals were visualized as either an increased number of *HER2 *signals, clusters of black dots with BT-474 tumor, and/or both (Figure 2B). Single CEN 17 copies were observed as red dots that were slightly larger than the black dots for *HER2 *genes in the nuclei with MCF7 tumor (Figure 2C) and BT-474 tumor (Figure 2D). After single staining for *HER2 *gene or CEN 17 was optimized, the BDISH application with sequential detection for HER2 targets followed by CEN 17 targets was tested on xenograft tumors. Single copies of *HER2 *genes and CEN 17 were stained in the nuclei of MCF7 tumor cells (Figure 2E) and amplified *HER2 *genes and single copies of CEN 17 were visualized in the nuclei of BT-474 tumor cells (Figure 2F). Because of the size difference and color contrast of black dots for *HER2 *gene and red dots for CEN 17, they could be visually separated even when red and black signals were co-localized in the nuclei of MCF7 tumor cells (arrowheads, Figure 2E). When CEN 17 probe was omitted from the complete BDISH assay, there was no fast red staining on xenograft tumor sections (data not shown). Thus, the anti-DNP antibody used for CEN 17 signal detection (the second ISH detection) didn't recognize the DNP-hapten of *HER2 *probe signal (the first ISH detection) even though the same hapten was used for the sequential hybridization method. For image comparison of BDISH and FISH for *HER2 *gene and CEN 17, FISH images with MCF7 tumor and BT-474 tumor are presented in Figure 2G and Figure 2H, respectively. *HER2 *genes are seen as red-orange dots and CEN 17 targets are seen as green dots.

One successful way to preserve the fast red staining was the use of a toluene-based Cytoseal 60 mounting medium placed onto completely dried tissue sections prior to coverslipping with glass coverslips. We also confirmed that the red signal was successfully preserved with the Tissue-Tek^® ^film coverslipper method (Sakura Finetek Japan, Tokyo, Japan) after air-drying slides (data not shown). The most common method of coverslipping tissue sections stained with fast red is the use of an aqueous mounting medium. However, this method did not produce crisp fast red staining for quantitative analyses of BDISH signals (data not shown). For the second color for the BDISH application, DAB, BCIP/NBT, and TMB detection systems that produce brown, blue, and green to blue final product, respectively, were evaluated. However, they did not provide sufficient contrast against the *HER2 *ISH black signal (data not shown).

### BDISH assay performance

After optimizing the BDISH assay for *HER2 *gene and CEN 17 with formalin-fixed, paraffin-embedded xenograft tumor sections, we applied the assay to 94 breast carcinoma cases and scoring BDISH slides was conducted by 4 observers (MK, MD, FPL, and RRT). The consensus among observers was defined as the agreement of three or more observers on a given observation. With the historical scoring method (Negative: *HER2*/CEN 17 < 2 and Positive: *HER2*/CEN 17 ≥ 2.0) (Table 1), the consensus concordance rate was 98.9% (Simple Kappa = 0.9736, 95% CI = 0.9222 – 1.0000), the sensitivity was 96.3%, and the specificity was 100%. Individual concordance ranges were between 97.8% (Simple Kappa = 0.9466, 95% CI = 0.8736 – 1.0000) and 100% (Simple Kappa = 01.0000, 95% CI = 1.0000 – 1.0000). With the ASCO/CAP scoring method (Negative: *HER2*/CEN 17 > 1.8, Equivocal: 1.8 ≤ *HER2*/CEN 17 ≤ 2.2, and Positive: *HER2*/CEN 17 > 2.2) (Table 2), the consensus concordance rate was 95.7% (Simple Kappa = 0.8993%, 95% CI = 0.8068 – 0.9919). Individual concordance ranges were between 92.5% (Simple Kappa = 0.8275, 95% CI = 0.7102 – 0.9448) and 95.7% (Simple Kappa = 0.9069, 95% CI = 0.8206 – 0.9933). With the ASCO/CAP scoring method without the FISH equivocal cases (Table 3), the consensus concordance rate was 100% (Simple Kappa = 1.0000%, 95% CI = 1.0000 – 1.0000). The sensitivity was 100% and the specificity was also 100%. Individual concordance ranges were between 97.7% (Simple Kappa = 0.9442, 95% CI = 0.8678 – 1.0000) and 100% (Simple Kappa = 1.0000, 95% CI = 1.0000 – 1.0000).

**Table 1:** Performance of brightfield double *in situ *hybridization (BDISH) with clinical samples based on the historic scoring method

		**FISH**		***Total***
			
		*Positive*	*Negative*	
**BDISH**	*Positive*	26	0	26
	*Negative*	1	66	67
	***Total***	27	66	93

Frequency missing = 1

Sensitivity 96.3%

Specificity 100%

Concordance 98.9%

Kappa 0.9736

**Table 2:** Performance of brightfield double *in situ *hybridization (BDISH) with clinical samples based on the ASCO/CAP method with FISH equivocal cases

		**FISH**			***Total***
			
		*Positive*	*Equivocal*	*Negative*	
**BDISH**	*Positive*	25	1	0	26
	*Equivocal*	0	0	0	0
	*Negative*	0	3	63	66
	***Total***	25	4	63	92

Frequency missing = 2

Concordance 95.7%

Kappa 0.8993

**Table 3:** Performance of brightfield double *in situ *hybridization (BDISH) with clinical samples based on the ASCO/CAP method without 4 FISH equivocal cases

		**FISH**		***Total***
			
		*Positive*	*Negative*	
**BDISH**	*Positive*	25	0	25
	*Negative*	0	63	63
	***Total***	25	63	88

Frequency missing = 2

Sensitivity 100%

Specificity 100%

Concordance 100%

Kappa 1.0000

**Figure 3:**
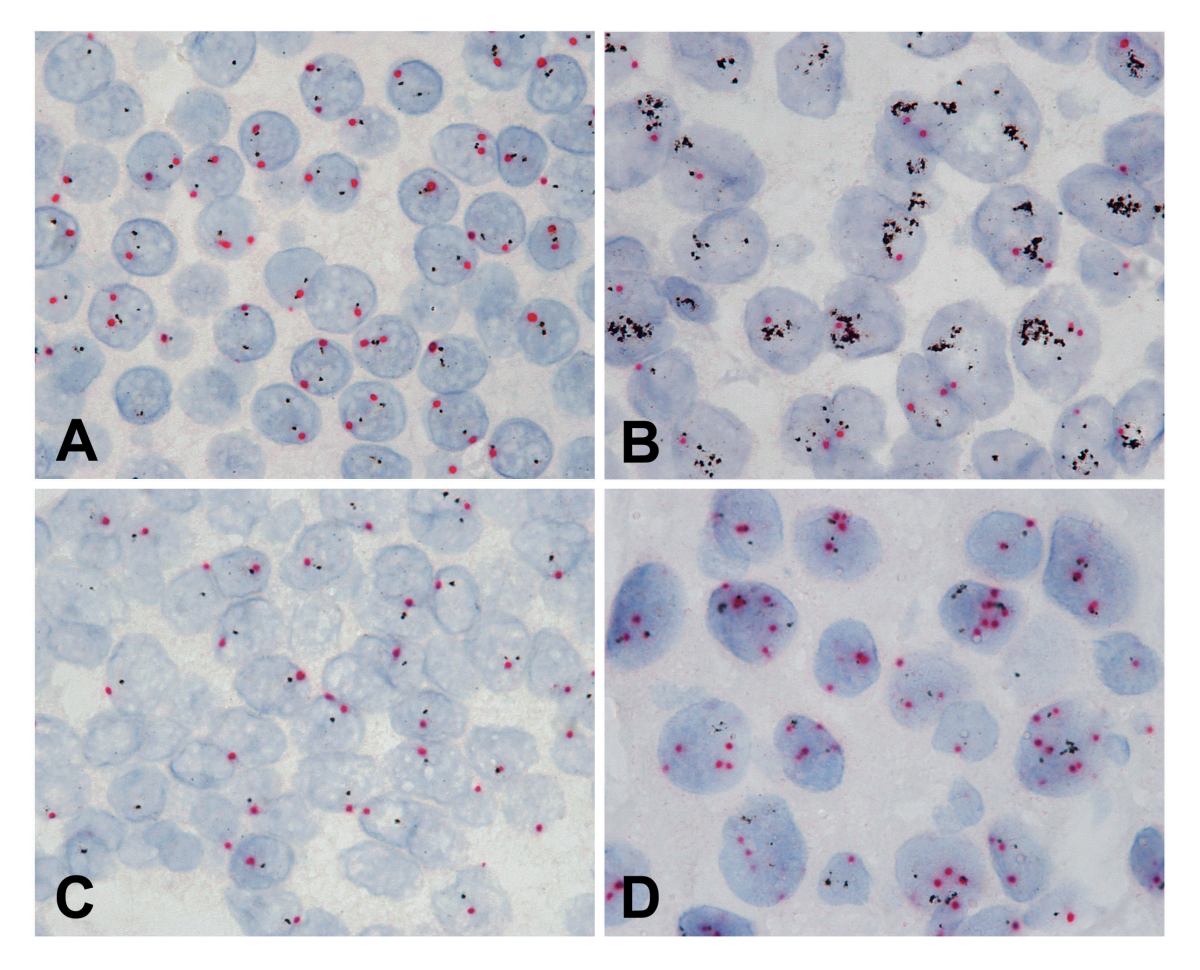
**Brightfield double *in situ *hybridization (BDISH) for *HER2 *and chromosome 17 centromere (CEN 17) on formalin-fixed, paraffin-embedded clinical breast cancer cases**. Examples of normal *HER2 *gene (A), amplified *HER2 *gene (B), single *HER2 *gene (C), and chromosome 17 polysomy (D) cases were shown. 100×.

Representative images of BDISH staining on clinical samples are presented in Figure 3. Cancer cells were easily identified based on the tissue morphology and assessments of *HER2*/CEN 17 ratios could be readily conducted. Non-amplified *HER2 *gene cases showed 0–4 copies of *HER2 *genes and 0–4 copies of CEN 17 depending on cell cycle stage and how each cell was cut within a tissue section (Figure 3A), while amplified *HER2 *gene cases showed multiple copies or clusters of *HER2 *genes and a few copies of CEN 17 (Figure 3B). Besides non-amplified and amplified *HER2 *cases, cases with a single copy of *HER2 *gene due to centromere 17 monosomy or a monoallelic deletion of *HER2 *gene (Figure 3C) and multiple copies of CEN 17 due to chromosome 17 polysomy (Figure 3D) were also observed.

**Figure 4:**
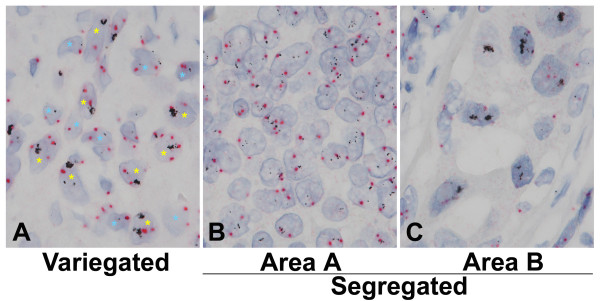
**The heterogeneity of breast tumor cell populations**. The heterogeneity of breast cancer cells was demonstrated with brightfield double *in situ *hybridization (BDISH) stained tissue sections. In general, there were 2 types of the tumor cell heterogeneity: 1) variegated tumor populations of, as an example, non-amplified (blue asterisk) and amplified (yellow asterisk) *HER2 *gene cells in the same area (A) and 2) segregated tumor populations of, as an example, discrete (B) and clustered (C) *HER2 *gene cells in different areas. The tumor cell heterogeneity with different appearance of *HER2 *and CEN 17 is also seen among the non-large clustered *HER2 *cells (B). 100×.

All discordant cases, that we defined it even by one observer disagreement between the BDISH and FISH scores, were re-examined for possible causes of conflicting results and there were 9 discordant cases. All nine (9) discordant cases of the BDISH slides presented at least some degree of the genotypic heterogeneity of tumor cell populations. In general, there were two types of the tumor cell heterogeneity with *HER2 *gene status within the same tissue section: 1) variegated different genotype tumor cell populations in the same area of tissue section (Figure 4A) and 2) segregated tumor populations in different areas of tissue section (Figures 4B&C). Breast cancers with obvious tumor cell heterogeneity are shown as examples in Figure 4. However, the subtle genotype heterogeneity of tumor cell populations is often seen among the equivocal cases, and it also can be seen in Figures 3D &4B which show less obvious variegated tumor cell heterogeneity. Three (3) of 9 discordant cases demonstrated the segregated tumor cell heterogeneity while the other 6 cases showed various degrees of the variegated tumor cell heterogeneity.

## Discussion

Accurate *HER2 *status testing is important for identifying breast cancer patients who may benefit from receiving trastuzumab therapy. Currently, in the United States, HER2 IHC methods are most commonly used for primary screening for *HER2 *status, and borderline cases are subjected to dual FISH for *HER2 *and CEN 17 to determine the *HER2*/CEN 17 ratio. Because the discordance rate between local and central/reference *HER2 *status testing with IHC and FISH is significantly high [14,31-33], the standardization of diagnosing breast cancer cases is recognized as a very important task for improving personalized cancer patient care [3,34]. The American Society of Clinical Oncology and the College of American Pathologists has published a guideline recommendation for testing *HER2 *status in breast cancer [3] and the Canadian National Consensus has updated the Canadian *HER2*/*neu *testing guideline [35]. Two potential solutions for improving the standardization of *HER2 *status testing include: 1) automating the entire process for slide staining [36] and slide reading [36-39] and 2) consolidating the *HER2 *testing process within experienced laboratories and pathologists that perform large numbers of *HER2 *tests [15].

One way to improve the accuracy of *HER2 *status testing is to automate the assay procedure for HER2 IHC and *HER2 *FISH assays so that human errors can be diminished. HER2 IHC assays can be performed using an automated slide staining system, but *HER2 *FISH assays remain technically challenging and time consuming manual molecular diagnostic assays in most laboratories. An evaluator of FISH slides must have access to specialized fluorescence microscopy in a dark room. Because of unstable FISH staining characteristics, the signals of FISH slides can be bleached easily, even while reviewing and enumerating signals. Furthermore, digital images of the FISH slide need to be captured with a sensitive camera system for each patient case for the *HER2 *gene status record. Therefore, it is desirable to automate a tissue-based *HER2 *gene status test that can be observed with a regular brightfield microscope and that produces stained slides that can be archived.

While the concept of multi-color brightfield ISH applications was published in 1990's [40,41], it was a recent achievement to visualize *HER2 *and CEN 17 targets within the same nuclei of tissue sections with a manual dual brightfield ISH application [42]. This dual ISH application utilized TMB chromogen for *HER2 *gene staining. However, based on published images [28,42], TMB staining does not provide discrete signals when compared to the SISH application. The advantages of the BDISH application for *HER2 *gene and CEN 17 presented in the current study are: 1) the automation of the ISH application; 2) the visualization of both *HER2 *gene and CEN 17 targets in the nuclei of the same cell; 3) the generation of discrete *HER2 *gene signals; 4) the ability to reproducibly detect endogenous *HER2 *and CEN 17 signals in the stromal tissues and lymphocytes as a reliable internal assay control; 5) the ability to visualise signal with brightfield microscopy with non-oil immersion lenses; and 6) the capability to permanently archive the slides.

*HER2 *and CEN 17 probes are co-hybridized for dual color HER2 FISH. However, for the BDISH assay, because the stringency conditions for the nick-translated *HER2 *probe and the CEN 17 oligoprobe were different, it was necessary to conduct sequential ISH staining steps for *HER2 *gene and CEN 17 targets. For CEN 17 ISH, we have optimized a new detection system with an alkaline phosphatase-conjugated antibody and fast red chromogen and naphthol phosphate substrate reaction. The fast red-based detection was selected to obtain a good contrast of CEN 17 ISH signal against the discrete black dots of *HER2 *SISH signal. DAB, BCIP/NBT, and TMB detection systems did not provide sufficient contrast against *HER2 *ISH black signal (data not shown). Because fast red precipitate is soluble in organic solvents, in general, aqueous mounting medium is used for coverslipping. However, the standard coverslipping method with aqueous mounting medium on wet tissue sections did not produce tissue sections with high resolution and therefore detailed tissue structure could not be observed (data not shown). A successful method to preserve fast red staining for CEN 17 and high resolution tissue morphology was, after completely dry the slides, to apply a toluene-based tissue mounting medium (Cytoseal 60) for coverslipping with cover glass or to use a film coverslipper (Tissue-Tek^® ^film coverslipper). Incomplete drying resulted in faint red background staining particularly around the fast red precipitate sites with this method. Interestingly, the use of aqueous mounting medium onto the dried tissue slides produced yellowish background staining on tissue sections and this method did not produce satisfactory results (data not shown).

The specificity of single ISH for *HER2 *gene or CEN 17 and BDISH for both targets was evaluated with xenograft tumors. *HER2 *and CEN 17 copy numbers have been documented previously using the FISH assay [29]. MCF-7 cells are characterized as non-amplified *HER2 *and chromosome 17 polysomy (3 copies of chromosome 17 per nucleus) while one of chromosome 17 with *HER2 *deletion (2 *HER2 *copies per nucleus) [29]. BT-474 cell line presents *HER2 *amplification with 50–60 copies of *HER2 *genes and 4–6 copies of CEN 17 per nucleus [29]. Amplified *HER2 *genes are located not only on chromosome 17, but also are translocated on other chromosomes [29]. *HER2 *and CEN 17 copy numbers produced with the single target ISH and BDISH methods matched with previously reported results. As both probes are labeled with the same DNP hapten, our first concern was to determine if detecting specific signal for each probe was feasible. We confirmed that the fast red chromogen detection reagents did not produce red signal when the fast red ISH was performed without the CEN 17 probe after detection of *HER2 *by SISH (data not shown). Thus, the SISH detection and the fast red detection can be combined to perform a sequential double ISH assay with 2 probes labeled with the same hapten. Because the sequential BDISH application uses 2 specific stringency conditions based on the length and sequences of 2 probes, it is not necessary to design 2 probes that require the same stringency for co-hybridization, like double color FISH assays.

Concordance rates between a set of gold standard dual color *HER2 *FISH scores and *HER2 *and CEN 17 BDISH scores by 4 observers were calculated for assessing the performance of the BDISH assay. There were 9 discordant cases (9.6% of the total cases) based on BDISH score disagreement with FISH scores, even by one observer. We have found that the number of equivocal cases influences the concordance rate with the ASCO/CAP scoring method. There were 4 equivocal cases based on FISH scores and all cases showed the BDISH score disagreement by at least 2 observers. A similar observation was reported with an international *HER2 *testing proficiency study [15]. In their study, the discordant cases (20%) were caused by the specimen having FISH *HER2*/CEN 17 ratios between 1.7 and 2.3 that are close to the 'equivocal' defined by ASCO/CAP *HER2 *scoring method (1.8 – 2.2). They also stated "equivocal cases are difficult to interpret, even highly experienced and validated laboratories" [15]. In one study, when the FISH assay was used as the primary test for *HER2 *status assessment of breast carcinoma cases, heterogeneity of *HER2 *gene status was observed in 40 of 742 cases (5%) [43]. It has been speculated that genomic and phenotypic heterogeneity of tumor cells is the main reason for the inconsistency of *HER2 *testing results [44]. With current study, all of our discordant cases (9/9 or 100%) displayed the tumor cell population heterogeneity: three samples showed significant segregated tumor cell population heterogeneity (Figures 4B&C) and other cases showed subtle heterogeneity of tumor cell populations that are seen among equivocal cases (4/4 or 100%). Nonetheless, since consecutive tissue sections were not used for the FISH and BDISH analyses, one can speculate that the tissue sections for the FISH and BDISH tissue sections contained tumor cell populations with different *HER2 *status. Further clinical evaluations of *HER2 *and CEN 17 BDISH application with patient treatment outcome data are required for more accurate *HER2 *status assessment of breast cancer patients to be obtained.

## Conclusion

We have successfully developed an automated BDISH application for *HER2 *gene and CEN 17 targets in formalin-fixed, paraffin-embedded tissue sections that is highly concordant to the FISH and is reproducibly interpreted among observers. Assessment of *HER2 *gene status can be conducted without the use of a specialized fluorescence microscope and the time required for completing *HER2 *gene status assessment can be shortened significantly. Furthermore, this application has the potential to be used for other gene targets, any combination of a gene and its chromosome centromere, and tissue section-based gene assessment tests including gene translocation studies. The use of BDISH technology allows the simultaneous analyses of two DNA targets within the context of tissue morphology observation.

## Competing interests

HN, BHW, ML, AEM, FG, MF, EW, TM are employed by Ventana Medical Systems, Inc. RRT and MD received grant support and honorarium for speaking from Ventana Medical Systems, Inc.

## Authors' contributions

LW, ML, JP, and RRT were responsible for identifying and prequalification of the clinical cases used in this study. HN and TMG were responsible for the BDISH assay development and feasibility studies, staining the clinical samples, and preparing the manuscript draft and image data. BHW and ML were responsible for the final assay development. AEM conducted all statistical analyses for the performance of BDISH assay. FG was the study coordinator and contributed intellectual content of the study. MF designed the probes. EW, MK, MD, RRT, and TMG critiqued the assay performance with their molecular histology expertise during the assay development. FPL, MK, MD, and RRT were the observers for scoring the clinical samples. All authors contributed intellectual inputs to the study. All authors read and approved the final manuscript.
